# Efficient lentivirus concentration using a tabletop centrifuge

**DOI:** 10.1007/s11033-026-12080-7

**Published:** 2026-06-09

**Authors:** Eva Müller, Julia Boesner, Zehra Karaköse, Roland Schwarzer, Hannah S. Schwarzer-Sperber

**Affiliations:** Institute for the Research on HIV and AIDS-associated Diseases, University Hospital Essen, University of Duisburg-Essen, Essen, Germany

**Keywords:** Lentivirus, Centrifugation, Virus concentration, Infection

## Abstract

**Background:**

Efficient virus concentration is a crucial prerequisite for many research applications including gene delivery using lentiviral vectors, antiviral drug screening, and studies of viral infection mechanisms. Typically, concentration methods involve the pelleting of viral particles and their resuspension in a defined medium to allow precise experimental control over the viral titer and media composition. However, commonly used techniques, such as ultracentrifugation, PEG precipitation or density gradient centrifugation, while effective, are limited by their fairly high cost, significant processing times, and limited availability.

**Results:**

Our study demonstrates that virus concentration using a tabletop centrifuge at 16,100 g yields higher virus titers and allows superior infection efficiencies compared to PEG precipitation-based methods. Furthermore, we optimized the protocol by reducing the centrifugation time to as little as 15 min, while maintaining effective virus enrichment.

**Conclusions:**

We demonstrate that a rapid 15-minute centrifugation using a tabletop centrifuge is sufficient for robust virus concentration. This streamlined approach enhances viral titers, enables efficient medium exchange, and offers a cost-effective and accessible alternative to conventional protocol, particularly valuable in high-biosafety-level laboratories where access to specialized equipment such as ultracentrifuges is often constrained.

**Supplementary Information:**

The online version contains supplementary material available at 10.1007/s11033-026-12080-7.

## Introduction

A crucial aspect of virus-based work is the viral titer, that is, the concentration of virus in a given preparation, which can be assessed by either functional infectivity or physical particle-based readouts. The ability to increase the viral titer by enriching the virus is of essential importance for various applications such as gene delivery using lentiviral particles, testing antiviral drugs, or investigating the mechanisms of infection [1, 2]. In most workflows, viral particles are pelleted and resuspended in a defined volume and medium, allowing adjustment of virus input and buffer conditions. Established concentration methods include ultracentrifugation, density gradient-based procedures, and Polyethylene Glycol (PEG) precipitation [3–11]. However, these approaches usually are cost- and time-intensive.

Here, we tested whether a standard tabletop centrifuge can provide an effective alternative for lentivirus concentration and medium exchange, and compared this approach with a widely used PEG-based method.

## Results

**Fig. 1:**
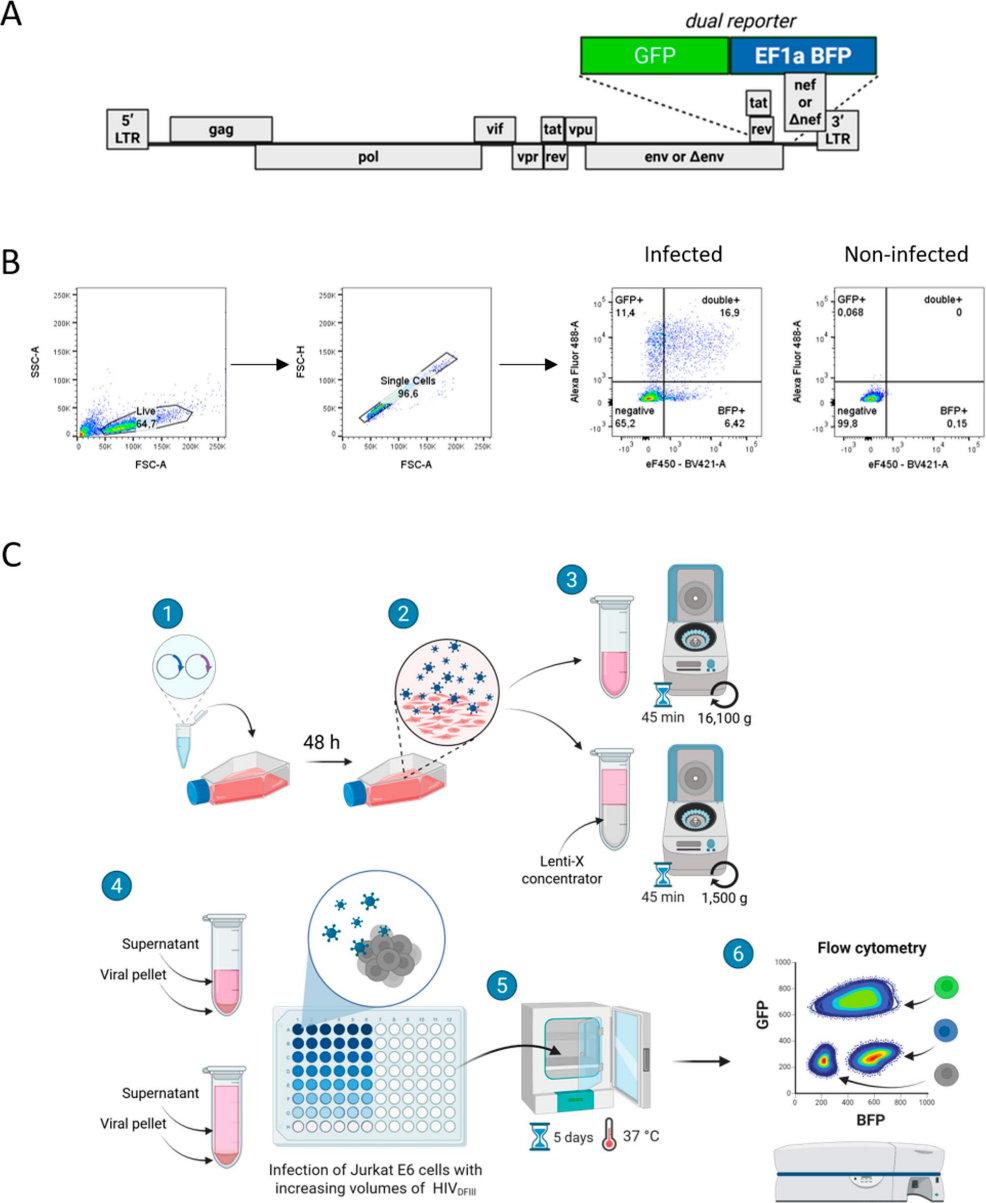
**Schematic representation of HIV**_DFIII_**production and subsequent infection of Jurkat E6 cells. (A)** Schematic illustration of the HIV_DFIII_ lentiviral construct consisting of 17.5 kbp. Viral genes are shown as white boxes, whereas the fluorescent reporter cassette is highlighted in blue and green. **(B)** Gating strategy used for flow cytometric analysis to identify infected Jurkat E6 cells. Live cells were gated based on the Forward scatter/Side Scatter values, followed by singlet gating. Green fluorescent protein (GFP) and Blue fluorescent protein (BFP) expressing cells among live singlets were considered infected. **(C)** Experimental workflow to evaluate the efficiency of HIV_DFIII_ virus pelleting using tabletop centrifugation at 16,100 × g or Lenti-X concentrator. (1) HEK293T cells were transfected with HIV_DFIII_ and VSVg plasmid. (2) After 48 h, the virus-containing supernatant was collected. (3) The supernatant was concentrated using a tabletop centrifuge at 16,100 × g or the Lenti-X concentrator system. (4) Infection of Jurkat E6 cells with titrated viral supernatant or concentrated HIV_DFIII_. (5) Incubation of infected Jurkat E6 cells for five days at 37 °C (6) Assessment of infection levels by flow cytometry.

The aim of this study was to establish a rapid, simple, and cost-effective method to concentrate lentivirus-containing solutions. To assess its efficacy, we compared it to a widely used commercial PEG-based precipitation concentrator. For this purpose, we produced lentiviral particles using a well-established, replication-deficient human immunodeficiency virus (HIV)-derived construct containing two fluorescent reporters (Fig. 1A). This viral construct, called HIV_DFIII_, is frequently used in HIV persistence research [12–14] and is particularly difficult to produce due to the large size of the modified lentiviral genome (> 17.5 kilo base pairs (kbp)). First, we produced HIV_DFIII_ in HEK293T cells and concentrated the virus-containing supernatant using a tabletop centrifuge at maximum speed (16,100 g) only or the PEG-based precipitation Lenti-X concentrator. PEG reduces viral solubility through a volume-exclusion effect, driving particles into closer proximity and facilitating their aggregation and recovery by centrifugation [15, 16]. The infectivity of increasing amounts of the resulting virus concentrate, as well as the supernatant, was then assessed in Jurkat E6 cells (Fig. 1C). Infection levels were quantified by measuring HIV_DFIII_ fluorescence expression by flow cytometry (Fig. 1B).

**Tabletop centrifugation achieves similar virus concentration compared to gradient-based methods.** To directly compare the performance of tabletop centrifugation with a commercial gradient-based approach, we concentrated virus-containing supernatants using both methods under matched conditions, each with a 45-minute centrifugation step. The non-concentrated virus served as an additional control. The efficiency of the concentration methods was assessed using both quantitative PCR (qPCR) to determine viral genome copy numbers and a functional infectivity assay as a biologically relevant measure of viral activity.

**Fig. 2:**
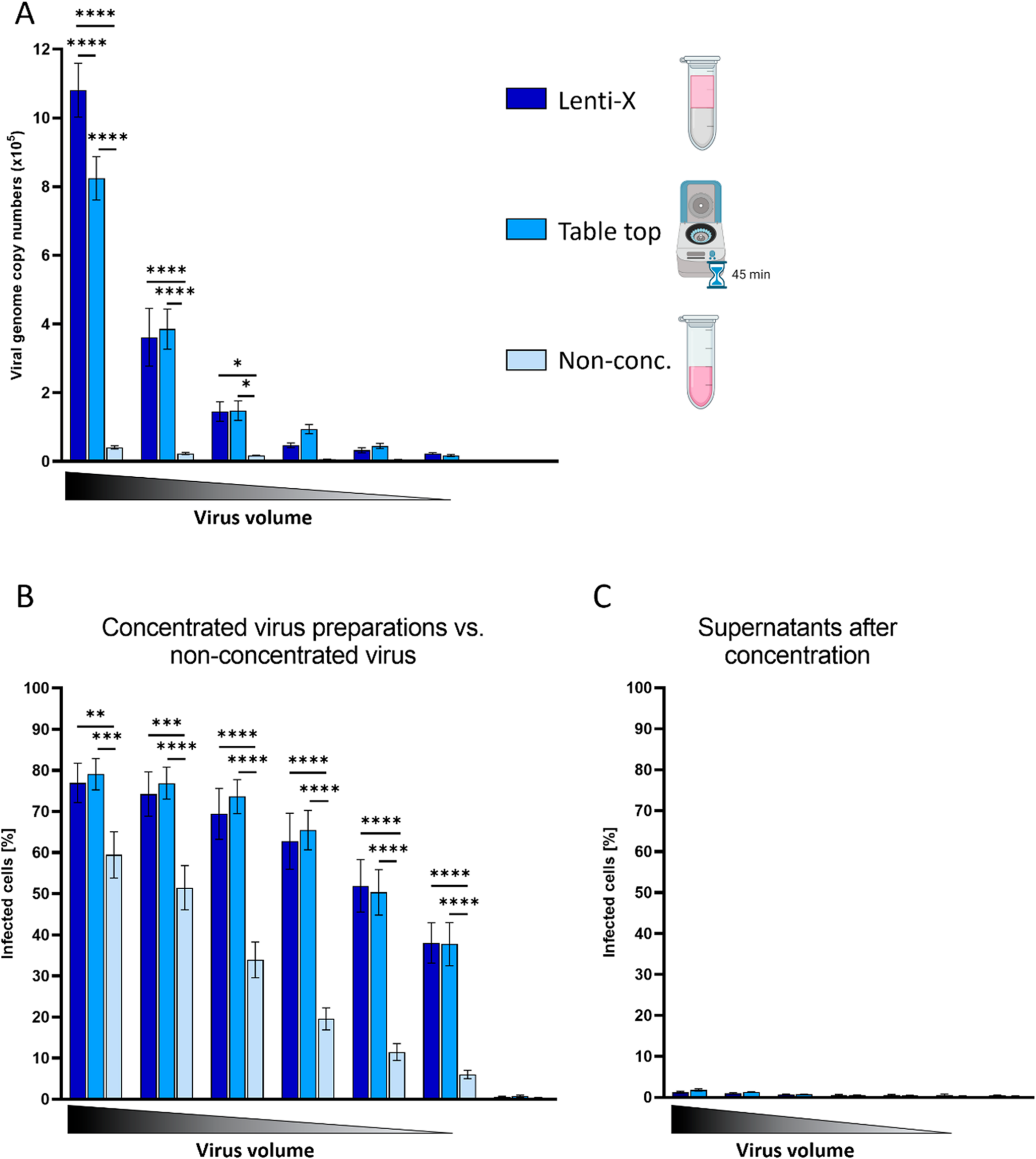
**Efficient virus concentration using a tabletop centrifuge.** HIV_DFIII_ virus was harvested and either left unconcentrated, concentrated by tabletop centrifugation, or concentrated with the Lenti-X reagent. All assays were performed using two-fold serial dilutions with a range from 50 µL to 1.6 µL. **(A)** Viral genome copy numbers were quantified by qPCR. **(B)** Jurkat E6 cells were infected with serial dilutions of concentrated and non-concentrated virus as comparison. **(C)** To assess enrichment efficiency, Jurkat E6 cells were infected for five days with serial dilutions of the supernatant remaining after virus pelleting. Panels B and C represent functional infectivity readouts measured by flow-cytometric quantification of GFP/BFP-positive Jurkat E6 cells after infection with serially diluted virus preparations. Cells were considered positive if expression of infection markers exceeded the background signal from non-infected controls. Bars represent mean with standard error of the mean (SEM). Significance was tested using Ordinary two-way Anova with Tukey’s multiple comparison test, ** *P* ≤ 0.01, *** *P* ≤ 0.001, **** *P* ≤ 0.0001, *N* = 10 from two independent experiments.

The qPCR revealed that both concentration methods significantly increased viral genome copies compared to the non-concentrated condition (Fig. 2A). The PEG-based precipitation with the Lenti-X concentrator yielded the highest number of viral genomes at the largest inoculum, whereas at smaller inocula both methods resulted in comparable copy numbers. To facilitate direct comparison of concentration efficiency, we also calculated HIV_DFIII_ genome recovery relative to the corresponding non-concentrated input aliquot based on viral genome copy number. Using an input volume within the linear range of the assay (25 µL), recovery was comparable between tabletop centrifugation (~ 111%) and Lenti-X (~ 104%).

For the functional infectivity assessment, we carried out a reporter-based infection assay in Jurkat E6 cells and quantified infected cells by flow cytometry (Fig. 2B). Both concentration methods significantly exceeded the infection levels of the non-concentrated virus, equally. Estimated functional titers (TU/mL) derived from low-transduction conditions are provided in Supp. Figure 1 and support the same overall conclusion. However, the increase in functional infectivity was less pronounced than the increase in viral genome copy numbers, indicating that concentration enriched genome-positive material more strongly than functionally infectious particles. To determine how effectively the viral particles were pelleted, we also tested the residual infectivity in the supernatants remaining after each concentration step (Fig. 2C). Importantly, only concentrated preparations generate both, a resuspended virus fraction and a post-concentration supernatant fraction; for non-concentrated virus, only the original input preparation was available and no corresponding supernatant fraction could be analyzed. Notably, none of the tested concentration methods left substantial infectivity in the supernatant.

Together, these results demonstrate that a 45-minute spin at 16,100 × g in a standard tabletop centrifuge is sufficient to concentrate lentivirus and can achieve yields and efficiencies comparable to those obtained with the widely used commercial method.

**Fig. 3:**
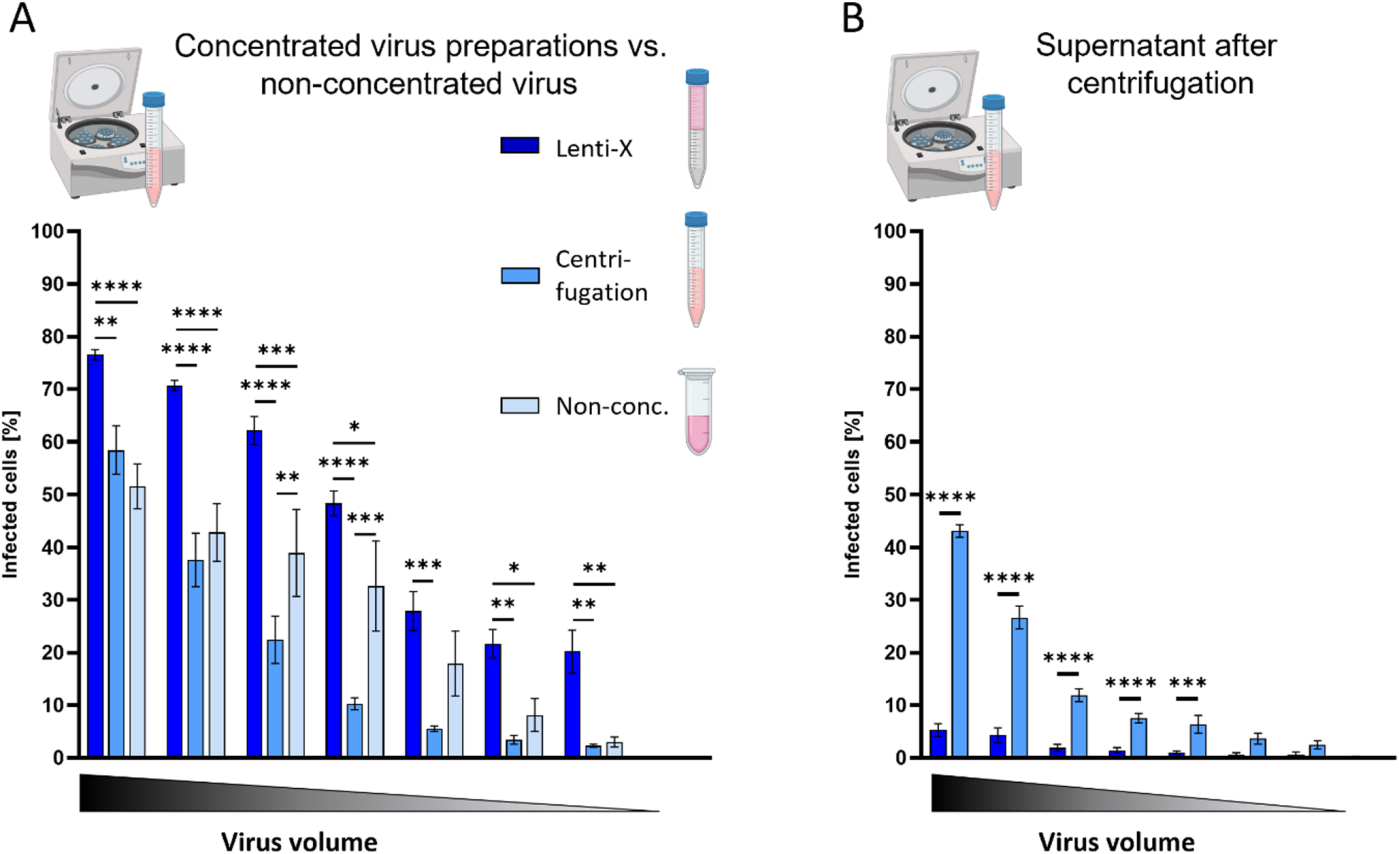
**Low-speed centrifugation is ineffective for concentrating lentivirus in large volumes.** HIV_DFIII_-containing supernatant was concentrated by pelleting either by centrifugation at 4,696 × g for 45 min or by using the PEG-precipitation-based Lenti-X Concentrator, followed by resuspension in cell culture medium. Two-fold serial dilutions of the resulting viral preparations were used to infect Jurkat E6 cells, with inoculum volumes ranging from 50 µL to 0.8 µL. **(A)** Infection with resuspended virus preparations obtained after concentration, shown in comparison with non-concentrated virus input. **(B)** Infection with the supernatants remaining after the concentration procedure. Infection efficiency was determined via flow cytometry, using non-infected cells to set the baseline threshold. Data are presented as mean ± SEM. Significance was tested using Ordinary two-way Anova with Tukey’s multiple comparison test, ** *P* ≤ 0.01, *** *P* ≤ 0.001, **** *P* ≤ 0.0001, *N* = 6.

**Low-speed centrifugation in large volumes does not efficiently enrich lentivirus preparations.** Having demonstrated that centrifugation at 16,100 × g effectively pellets viral particles, we next sought to determine whether centrifugation of larger volumes in 15 mL tubes at a lower speed (4,696 × g) would also be effective. To compare virus titers, HIV_DFIII_ preparations concentrated solely at 4,696 × g, via PEG-precipitation-based concentrator Lenti-X, or left unconcentrated were titrated on Jurkat E6 cells (Fig. 3).

The results showed that centrifugation at 4,696 × g was not effective for virus concentration. Infection rates were similar to or even significantly lower than those observed with non-concentrated virus, particularly upon low viral input. Here, in contrast to the previous high velocity centrifugation, PEG-precipitation-based concentration achieved significantly higher infection rates compared to centrifugation at 4,696 × g or non-concentrated virus.

The evaluation of the viral supernatant collected after the concentration process confirmed these findings; centrifugation at 4,696 × g, without the use of a gradient, did not efficiently pellet viral particles (Fig. 3B).

**Fig. 4:**
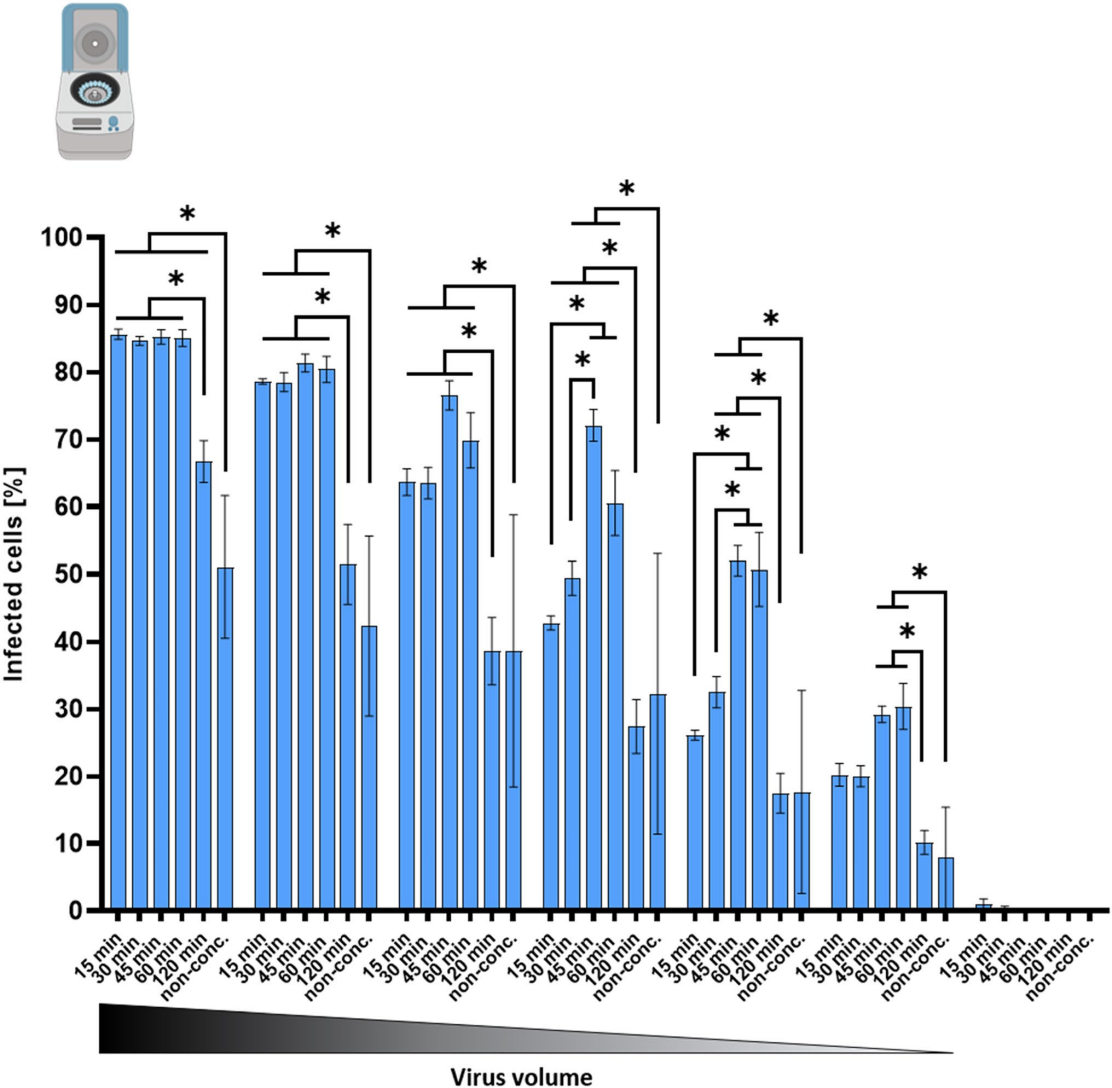
**A 15-minute tabletop centrifugation is sufficient to pellet lentiviral particles.** HIV_DFIII_-containing supernatant was concentrated by pelleting using a tabletop centrifuge at 16,100 × g for the indicated time periods, followed by resuspension in cell culture medium. Two-fold serial dilutions of the resulting viral preparation were used to infect Jurkat E6 cells, with inoculum volumes ranging from 50 µL to 1.6 µL. Infection rates were measured by flow cytometry based on expression of infection markers, with non-infected cells defining the threshold for positive signal. Data are presented as mean ± SEM. Significance was tested using Ordinary two-way Anova with Tukey’s multiple comparison test, * *P* ≤ 0.05, *N* = 6.

**Tabletop centrifugation of 15 min can pellet viral particles effectively.** To further optimize viral pelleting by high-speed tabletop centrifugation, 1.5 mL aliquots of viral supernatant were centrifuged at 16,100 × g for 15, 30, 45, 60, and 120 min and compared to non-concentrated controls (Fig. 4). The resulting virus preparations were then tested in Jurkat E6 cells using two-fold serial inoculum volumes ranging from 50 µL to 1.6 µL. Remarkably, even the shortest centrifugation time of 15 min did not reduce the maximum infection rate for larger inocula (50 and 25 µL). In fact, centrifugation periods between 15 and 60 min resulted in nearly a two-fold increase in maximum infectivity compared to non-concentrated virus. At inocula equal to or lower as 12.5 µL, the highest infection rates were observed using virus centrifuged for 45 and 60 min. By contrast, extending the centrifugation to 120 min reduced infectivity rates to levels comparable to non-concentrated virus (Fig. 4). In summary, tabletop centrifugation time can be lowered to as little as 15 min, providing a rapid and effective method to pellet viral particles and significantly enhance infectivity.

## Discussion

**Fig. 5:**
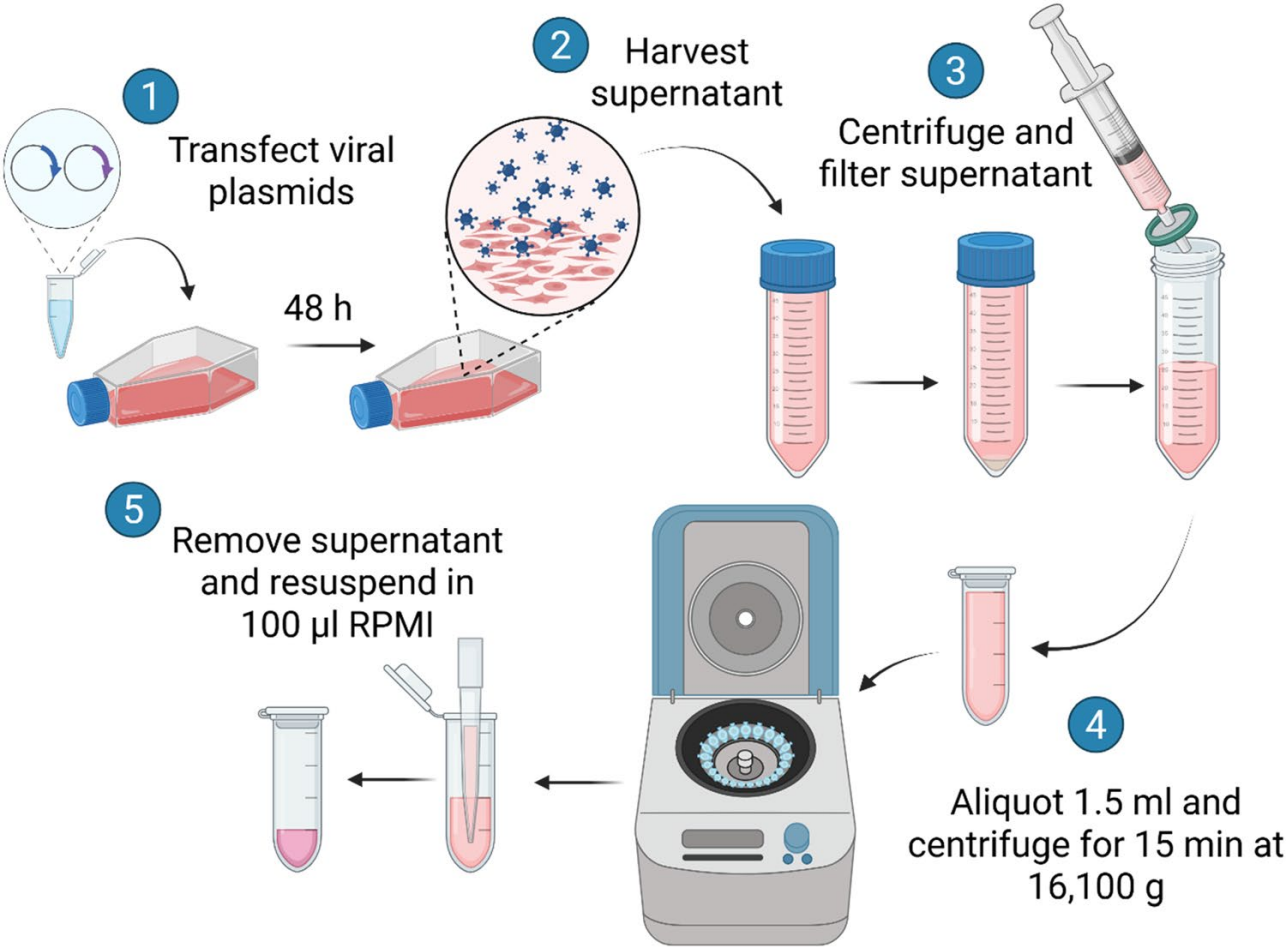
**Schematic workflow of virus production and concentration using a tabletop centrifuge.** (1) HEK293T cells were transfected with HIV_DFIII_ and VSVg plasmid. (2) After 48 h, the virus-containing supernatant was collected. (3) The supernatant was centrifuged and filtered to remove cell debris. (4) Aliquots of 1.5 mL were transferred to reaction vessels and spun at 16,100 × g for 15 min. (5) The supernatant was removed, and the viral pellet was resuspended in 100 µL RPMI.

In this study, we present a simple, rapid, and cost-effective method for virus concentration using a standard tabletop centrifuge (Fig. 5). Our results show that virus concentrated by this approach achieves infection levels comparable to those obtained with PEG-based precipitation concentration (Fig. 2B). This is supported by genome quantification (Fig. 2A) and recovery analysis (~ 100% genome recovery), both indicating similar performance between the two methods. Notably, we further streamlined the process by reducing the centrifugation time to as little as 15 min (Fig. 4). In contrast, prolonged centrifugation of 120 min reduced functional virus yield compared to shorter centrifugation times (Fig. 4) indicating that extended processing may negatively affect functional virus yield. A centrifugation time of 45 min appears to provide an optimal balance between enrichment and preservation of infectivity.

While both concentration methods markedly increased viral genome copy numbers (Fig. 2A), the corresponding increase in functional infectivity was less pronounced (Fig. 2B). This difference should be interpreted with caution, because the two readouts capture different aspects of the virus preparation. qPCR measures genome-positive viral material and can increase proportionally with input, whereas the flow-cytometric infection assay reports the percentage of infected target cells and is inherently capped once a large fraction of susceptible cells has been infected. Accordingly, the infectivity readout is expected to plateau at high virus input and therefore does not scale linearly across the full concentration range. Beyond this assay-related limitation, the remaining discrepancy between genome copy number and functional infectivity may reflect the presence of non-infectious particles and may also be influenced by aggregate formation during concentration. However, aggregation was not directly measured in this study. In addition, properties of the viral construct itself may also influence the relationship between genome copy number and functional infectivity. The HIV_DFIII_ construct used in this work for virus generation is a large, replication-deficient reporter construct, which may generally limit production efficiency compared with smaller lentiviral vectors. However, in the non-concentrated preparation, PCR-based and functional infectivity titers were comparable, indicating that the HIV construct retains substantial infectivity.

This divergence between genome copy numbers and functional infectivity is relevant for downstream applications, as preparations normalized only by genome copy number may overestimate the amount of functionally active virus, particularly in comparative infectivity experiments. Functional virus readouts should therefore be considered alongside physical quantification when concentrated lentiviral preparations are used for infection-based studies.

Our data also show that centrifugation parameters matter. High-speed centrifugation in small tubes was effective (Fig. 2), whereas lower-speed centrifugation of larger volumes at 4,696 × g did not improve functional infectivity over non-concentrated virus under the tested conditions (Fig. 3). This indicates that simple transfer of the workflow to larger vessel formats is not sufficient unless adequate centrifugal force can still be achieved.

Several alternative virus concentration methods have been described, including ultracentrifugation [5, 6, 9], ion-exchange high-performance liquid chromatography (HPLC) [17], PEG precipitation [8], and gradient-based centrifugation [3, 4, 7]. Although ultracentrifugation is well established and yields high virus concentrations, it requires expensive, specialized equipment that is not available in many laboratories. Additionally, the handling of many viruses requires strict biosafety measures, which confine infectious procedures to designated containment laboratories. As a result, separate ultracentrifuges are often needed for infectious and non-infectious materials, further increasing the logistical and financial burden on laboratories and scientific institutes. HPLC, density gradient centrifugation, and PEG precipitation do not require an ultracentrifuge and offer the added advantage of separating cell debris from viral particles However, they often require additional expensive reagents and consumables. A common limitation among these conventional methods is their time-consuming nature. For example, ultracentrifugation typically requires around two hours [18], and gradient-based centrifugation at lower g-forces may take anywhere from 45 min to four hours [3].

On the contrary, our method is both highly accessible and time efficient. By employing a standard tabletop centrifuge and a brief 15-minute spin, we provide a practical and rapid alternative for virus concentration and straightforward exchange of virus media.

One limitation of this study is that the workflow was evaluated using a single large lentiviral construct (HIV_DFIII_ > 17.5 kbp). Because vector genome size can influence lentiviral production and functional yield, the extent to which the same concentration protocol performs similarly for lentiviral vectors carrying smaller transgenes was not systematically addressed here. This should therefore be evaluated in future studies using additional lentiviral backbones and transgene sizes. Additionally, post-concentration steps, such as filtration to reduce potential aggregates, could be explored to further improve functional virus yield. More broadly, the practical utility of centrifuge-based virus-handling workflows across experimental systems is supported by our recent Orthohantavirus methodology study, which established a standardized stock-generation and quantitative analysis pipeline without ultracentrifugation [19].”

## Materials and methods

*Cell culture*: Jurkat E6 and HEK293T cells were obtained from ATCC and maintained under standard cell culture conditions at 37 °C in a humidified atmosphere containing 5% CO₂. Jurkat E6 cells were cultured in Roswell Park Memorial Institute (RPMI) 1640 medium (Gibco, USA) supplemented with 10% (v/v) fetal bovine serum (FBS; Gibco, USA), 2 mM L-glutamine (Gibco, USA), and 1% (v/v) penicillin-streptomycin (Pen-Strep; Gibco, USA). HEK293T cells were cultured in Dulbecco’s Modified Eagle Medium (DMEM; Gibco, USA) with the same supplements: 10% (v/v) FBS, 2 mM L-glutamine, and 1% (v/v) Pen-Strep. Jurkat E6 and HEK293T cells were passaged at a 1:10 ratio twice a week, following a three- and four-day interval schedule.

*HIV*_*DFIII*_ *virus preparation*: HIV_DFIII_, a derivative of HIV_DFII_ originally generated and described in [14], is an Env-deficient, single-round, recombinant HIV construct that comprises two independent infection markers, BFP and GFP, and is often used for HIV reservoir and latency studies [12–14]. BFP expression is driven by the constitutive EF1α promoter, and cells expressing only BFP are considered latently infected. In contrast, GFP is expressed from the viral LTR, such that productively infected cells are identified by co-expression of GFP and BFP. To produce HIV_DFIII_ 14 × 10^6^ HEK293T cells were seeded the day before transfection in a T175 cell culture flask (Sarstedt, Germany). The following day, cells were transfected with 30 ug of total DNA, comprising the viral construct HIV_DFIII_ and the envelope plasmid pVSVg (Cat. # 12259, Addgene, USA) in a 1.73:1 ratio. Polyethyleneimine (PEI) (1 µg/µL, Polyscience, USA) was used as a transfection reagent in a 3:1 (PEI: DNA) ratio. DNA and PEI were diluted in optimized Minimal Essential Medium (Gibco, USA), mixed, and incubated for 15 min before being added to cells in antibiotic-free DMEM. At 24 h post-transfection, the medium was replaced with DMEM (Gibco, USA) containing antibiotics and serum. Cell culture supernatant containing viral particles was harvested 48 h post-transfection, centrifuged at 400 × g for 5 min to remove cell culture debris and filtered through a 0.22 μm filter (Avantor, USA). Because lentiviral particles (145 ± 25 nm [20]), are substantially smaller than 0.22 μm, passage through this filter is possible; however, such filtration may reduce viral recovery and was used here primarily to remove cellular debris before aliquoting. Importantly, the same pre-filtration step was applied to all virus preparations before any concentration procedure, so it does not account for the differences observed between non-concentrated, centrifuged, and Lenti-X-treated samples. Subsequently, the virus was aliquoted into 1.5 mL volumes for the concentration process and storage at -80 °C.

*Virus Concentration using the Lenti-X concentrator*: The virus was concentrated as described by the manufacturer. Shortly, the Lenti-X concentrator (Cat. #631231, Takara, Japan) was mixed with the virus solution in a 1:4 ratio and incubated for 45 min at 4 °C followed by centrifugation at 1,500 × g for 45 min at 4 °C. The supernatant was removed and the virus was resuspended in supplemented RPMI (Gibco, USA). Virus aliquots were frozen and stored at -80 °C.

*Virus Concentration using a tabletop centrifuge*: The virus preparations were spun in a table top centrifuge (Eppendorf, Germany) at 4 °C for the indicated time intervals at 16,100 × g. Subsequently, the supernatant was removed using a 1000 µL micropipette and the pellet was resuspended in supplemented RPMI (Gibco, USA). Virus aliquots were stored at -80 °C.

*Quantification of viral genome copy numbers*: HIV_DFIII_ virus dilutions were generated starting with 100 µL of undiluted virus. Subsequent two-fold dilutions were prepared by transferring 50 µL of the previous dilution into 50 µL RPMI. Viral RNA was extracted from each dilution using the QIAamp Viral RNA Mini Kit (Qiagen, Germany) according to the manufacturer’s instructions, except that carrier RNA was omitted. First-strand cDNA synthesis was performed with the HiScript III 1st Strand cDNA Synthesis Kit (Vazyme China). Quantitative PCR was carried out using Luna Universal qPCR Master Mix (New England Biolabs, USA) with primers targeting the HIV gag gene (Forward Primer: CATGTTTTCAGCATTATCAGAAGGA, Revers Primer: TGCTTGATGTCCCCCCACT). A standard curve prepared from serial dilutions of the DFIII plasmid was run along with the samples and used to convert Ct values to genome copy number. To enable direct comparison between concentration methods, HIV_DFIII_ recovery was calculated from qPCR-based viral genome copy numbers as the total genome copies in the resuspended concentrated preparation (C_conc_) relative to the total genome copies in the corresponding non-concentrated (C_non−conc_) input aliquot.

$$\:Recovery\:\left(\%\right)=\frac{{C}_{conc}/\mu\:L}{{C}_{non-conc}/\mu\:L}x\frac{{Volume}_{resusp}}{{Volume}_{start}}\times\:100$$

The starting virus volume subjected to concentration was 1.5 mL and the final resuspension volume was 100 µL.

*Jurkat E6 infection*: Jurkat E6 cells were infected with HIV_DFIII_ virus preparations concentrated via PEG precipitation, tabletop centrifugation, or left non-concentrated, as well as with supernatants from centrifuged samples. For each infection experiment, 100,000 cells per well were seeded in 96-well U-bottom plates (Sarstedt, Germany). HIV_DFIII_ virus dilutions were generated starting with 100 µL of virus diluted in 100 µL RPMI, leading to an effective virus volume of 50 uL. Subsequent two-fold dilutions were prepared by transferring 100 µL of the previous dilution into 100 µL RPMI. Cell culture medium was replaced with 100 µL of serially diluted virus solution. Plates were then centrifuged at 990 × g for 2 h at 37 °C to facilitate infection, followed by incubation at 37 °C with 5% CO₂ for five days. Sixteen hours post-infection, the medium was replaced with supplemented RPMI for the first time and subsequently refreshed every two days.

*Flow cytometry*: The cells were washed twice with PBS and resuspended in PBS for flow cytometry using a BD LSRII (BD, USA). The data were analyzed using FlowJo™ v10.8.1 (BD Life Sciences, USA). Cells were considered infected when fluorescence from either infection marker (GFP or BFP) exceeded the background threshold established with non-infected controls.

*Data visualization*: The data presented was visualized using GraphPad PRISM 8.4.3 for Windows (GraphPad Software, USA, www.graphpad.com). The bars show the means with SEM. Functional infectivity was assessed by flow-cytometric quantification of GFP- and/or BFP-positive Jurkat E6 cells after infection with serially diluted virus preparations. Because HIV_DFIII_ is an Env-deficient, single-round reporter virus, we used this reporter-based infectivity assay rather than a classical CPE-based TCID50 assay. This provided a direct functional readout of successful infection across the tested virus dilutions. Because this assay is bounded by the fraction of infectable target cells, the signal is expected to plateau at high virus input and should therefore not be interpreted as linearly proportional to viral genome copy number across the full titration range. In addition, estimated transduction units per mL (TU/mL) were calculated from low-transduction conditions (1.56 µL viral input per 10,000 target cells) only and are provided as a supplementary analysis.

## Supplementary Information

Below is the link to the electronic supplementary material.

Supplementary Material 1

## Data Availability

All data generated or analyzed during this study are included in this published article.

## References

[1] Dong W, Kantor B (2021) Lentiviral vectors for delivery of gene-editing systems based on CRISPR/Cas: Current state and perspectives. Viruses 13(7):7. 10.3390/v13071288 10.3390/v13071288 PMC8310029 34372494

[2] Kafri T (2001) Lentivirus vectors: difficulties and hopes before clinical trials. Curr Opin Mol Ther 3(4):316–326 PubMed PMID: 11525555 11525555

[3] Jiang W, Hua R, Wei M, Li C, Qiu Z, Yang X et al (2015) An optimized method for high-titer lentivirus preparations without ultracentrifugation. 10.1038/srep13875 10.1038/srep13875 PMC4562269 26348152

[4] McCombs RM, Rawls WE (1968) Density gradient centrifugation of rubella virus. J Virol 2(5):409–414. 10.1128/jvi.2.5.409-414.1968 4972151 10.1128/jvi.2.5.409-414.1968 PMC375628

[5] Cliver DO, Yeatman J (1965) Ultracentrifugation in the concentration and detection of enteroviruses. Appl Microbiol 13(3):387–392. 10.1128/am.13.3.387-392.1965 14325278 10.1128/am.13.3.387-392.1965 PMC1058261

[6] Prata C, Ribeiro A, Cunha Â, Gomes M, Almeida NC (2012) Ultracentrifugation as a direct method to concentrate viruses in environmental waters: virus-like particle enumeration as a new approach to determine the efficiency of recovery. J Environ Monit 14(1):64–70. 10.1039/C1EM10603A 22113738 10.1039/c1em10603a

[7] Reimer CB, Baker RS, vanFrank RM, Newlin TE, Cline GB, Anderson NG (1967) Purification of large quantities of influenza virus by density gradient centrifugation. J Virol 1(6):1207–1216. 10.1128/jvi.1.6.1207-1216.1967 5621490 10.1128/jvi.1.6.1207-1216.1967 PMC375411

[8] Kohno T, Mohan S, Goto T, Morita C, Nakano T, Hong W et al (2002) A new improved method for the concentration of HIV-1 infective particles. J Virol Methods 106(2):167–173. 10.1016/s0166-0934(02)00162-3 PubMed PMID: 12393147 12393147 10.1016/s0166-0934(02)00162-3

[9] Friedewald WF, Pickels EG (1994) Centrifugation and ultrafiltration studies on allantoic fluid, preparations of influenza virus. J Exp Med 79(3):301–317. 10.1084/jem.79.3.301 19871372 10.1084/jem.79.3.301 PMC2135374

[10] Sapula SA, Whittall JJ, Pandopulos AJ, Gerber C, Venter H (2021) An optimized and robust PEG precipitation method for detection of SARS-CoV-2 in wastewater. Sci Total Environ 785:147270. 10.1016/j.scitotenv.2021.147270 33940413 10.1016/j.scitotenv.2021.147270 PMC8086323

[11] Lewis GD, Metcalf TG (1988) Polyethylene glycol precipitation for recovery of pathogenic viruses, including hepatitis A virus and human rotavirus, from oyster, water, and sediment samples. Appl Environ Microbiol 54(8):1983–1988. 10.1128/aem.54.8.1983-1988.1988 2845860 10.1128/aem.54.8.1983-1988.1988 PMC202790

[12] Calvanese V, Chavez L, Laurent T, Ding S, Verdin E (2013) Dual-color HIV reporters trace a population of latently infected cells and enable their purification. Virology 446(1–2):283–292. 10.1016/j.virol.2013.07.037 24074592 10.1016/j.virol.2013.07.037 PMC4019006

[13] Chavez L, Calvanese V, Verdin E (2015) HIV latency is established directly and early in both resting and activated primary CD4 T cells. PLoS Pathog 11(6):e1004955. 10.1371/journal.ppat.1004955 PubMed PMID: 26067822; PubMed Central PMCID: PMC4466167 26067822 10.1371/journal.ppat.1004955 PMC4466167

[14] Sperber HS, Raymond KA, Bouzidi MS, Ma T, Valdebenito S, Eugenin EA et al (2023) The hypoxia-regulated ectonucleotidase CD73 is a host determinant of HIV latency. Cell Rep 42(11):113285. 10.1016/j.celrep.2023.113285 37910505 10.1016/j.celrep.2023.113285 PMC10838153

[15] Atha DH, Ingham KC (1981) Mechanism of precipitation of proteins by polyethylene glycols. Analysis in terms of excluded volume. J Biol Chem 256(23):12108–12117. 10.1016/S0021-9258(18)43240-1 7298647

[16] Hönig W, Kula MR (1976) Selectivity of protein precipitation with polyethylene glycol fractions of various molecular weights. Anal Biochem 72(1–2):502–512. 10.1016/0003-2697(76)90560-1 942070 10.1016/0003-2697(76)90560-1

[17] Yamada K, McCarty DM, Madden VJ, Walsh CE (2003) Lentivirus vector purification using anion exchange HPLC leads to improved gene transfer. Biotechniques 34(5):1074–1078. 10.2144/03345dd04 PubMed PMID: 12765034 12765034 10.2144/03345dd04

[18] Tiscornia G, Singer O, Verma IM (2006) Production and purification of lentiviral vectors. Nat Protoc 1(1):241–245. 10.1038/nprot.2006.37 17406239 10.1038/nprot.2006.37

[19] Schwarzer-Sperber HS, Mussfeldt T, Boesner J, Dluzak T, Sutter K, Schwarzer R (2026) A Standardized workflow for Orthohantavirus production, detection, and antiviral screening. Virol J 23(1):94. 10.1186/s12985-026-03136-y 41864935 10.1186/s12985-026-03136-y PMC13064277

[20] Briggs JAG, Wilk T, Welker R, Kräusslich H, Fuller SD (2003) Structural organization of authentic, mature HIV-1 virions and cores. EMBO J 22(7):1707–1715. 10.1093/emboj/cdg143 12660176 10.1093/emboj/cdg143 PMC152888

