# Supplementary material for: Efficient lentivirus concentration using a tabletop centrifuge
Source: Mol Biol Rep. 2026 Jun 9;53(1):913. doi: 10.1007/s11033-026-12080-7 (PMC13249748; doi:10.1007/s11033-026-12080-7)
Supplement: Supplementary file 1 — Supplementary Material 1 [file 11033_2026_12080_MOESM1_ESM.docx]

**Efficient Lentivirus Concentration Using a Tabletop Centrifuge**

**Supplement:**


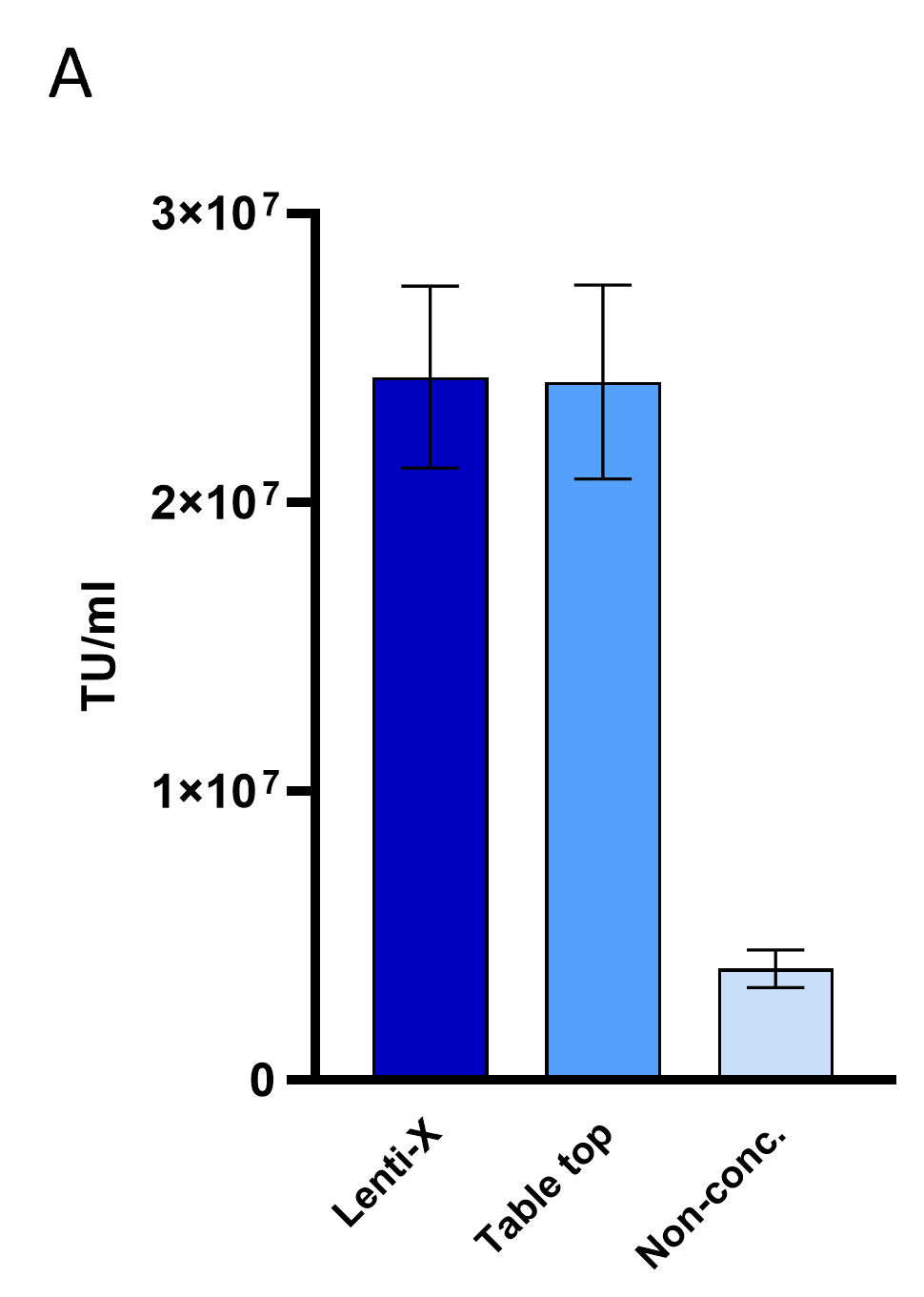


**Supp. Figure 1: Table-top concentration yields similar transduction units per mL as Lenti-X concentration.** Estimated lentiviral titers, presented here as transduction units per mL (TU/mL), were derived from the infection experiment shown in Figure 2. Titers were calculated based on the fraction of infected cells, 10,000 target cells, and a virus input volume of 1.56 µL. Bars represent mean values ± SEM.
